# *Mix* contraceptivo e fatores associados ao tipo de método usado pelas mulheres brasileiras: estudo transversal de base populacional 

**DOI:** 10.1590/0102-311XPT229322

**Published:** 2023-10-09

**Authors:** Fernanda Gontijo Araújo, Mery Nataly Silva Abreu, Mariana Santos Felisbino-Mendes

**Affiliations:** 1 Escola de Enfermagem, Universidade Federal de Minas Gerais, Belo Horizonte, Brasil.

**Keywords:** Anticoncepção, Saúde Reprodutiva, Saúde da Mulher, Iniquidades em Saúde, Estudos Epidemiológicos, Contraception, Reproductive Health, Women’s Health, Health Inequities, Epidemiologic Studies, Anticoncepción, Salud Reproductiva, Salud de la Mujer, Inequidades en Salud, Estudios Epidemiológicos

## Abstract

O objetivo deste estudo é descrever o *mix* contraceptivo e analisar os fatores associados ao tipo de contraceptivo usado pelas mulheres brasileiras em idade reprodutiva. Trata-se de estudo transversal, de base populacional, com dados de 19.962 mulheres de 15 a 49 anos. Os desfechos foram uso e tipo de contraceptivo, classificados em: contraceptivos reversíveis de curta duração (SARC), longa duração (LARC) e permanentes. As variáveis explicativas foram: características da história reprodutiva, sociodemográficas e de acesso aos serviços de saúde. Utilizou-se a regressão logística multinomial para estimativas da *odds ratio* (OR), tendo os SARC como categoria de referência. As análises foram realizadas no módulo *survey* do software Stata, que considerou o efeito do plano amostral complexo da *Pesquisa Nacional de Saúde* de 2019. A prevalência do uso de contraceptivos foi de 83,7%. Do total de usuárias, 72% usavam SARC, 23,2% métodos permanentes e 4,8%, LARC. Mulheres com maior escolaridade, plano de saúde, que tiveram partos e participaram de grupos de planejamento reprodutivo tiveram maior chance de usar LARC na comparação com o uso de SARC, enquanto o cadastro na unidade básica de saúde se associou a menor chance de uso. Ainda, quanto maior a idade e paridade, além de viver com o companheiro, maior a chance de usar métodos permanentes em relação ao uso de SARC. Apesar da elevada cobertura de contracepção, o *mix* contraceptivo permanece obsoleto, com predomínio do uso de SARC. Além disso, observou-se importante desigualdade de acesso, sendo os LARC acessíveis apenas por mulheres com melhores condições socioeconômicas, enquanto os métodos permanentes foram associados a um perfil de maior vulnerabilidade social.

## Abstract (en)

This study aims to describe the contraceptive mix and analyze the factors associated with the type of contraceptive used by Brazilian women of reproductive age. This is a cross-sectional, population-based study with data from 19,962 women aged 15 to 49 years. The outcomes were use and type of contraceptive, classified as: short-acting reversible contraceptives (SARC), long-acting (LARC), and permanent. The explanatory variables were characteristics of reproductive history, sociodemographic history, and access to health services. Multinomial logistic regression was used for odds ratio (OR) estimates, with SARC being the reference category. The analyses were performed in the Survey module of the Stata software, which considered the effect of the complex sampling plan of the 2019 *Brazilian National Health Survey*. The prevalence of contraceptive use was 83.7%. Of the total number of users, 72% used SARC, 23.2% permanent methods, and 4.8% LARC. Women with higher education, health insurance, who had deliveries, and who participated in reproductive planning groups had a higher chance of using LARC when compared with the use of SARC, while registration at the basic health unit was associated with a lower chance of use. Still, the higher the age and parity, in addition to living with the partner, the greater the chance of using permanent methods in relation to the use of SARC. Despite the high coverage of contraception, the contraceptive mix remains obsolete, with a predominance of the use of SARC. In addition, important inequalities in access were observed, with LARC being accessible only to women with better socioeconomic conditions, while permanent methods were associated with a profile of greater social vulnerability.

## Abstract (es)

El objetivo fue describir la combinación anticonceptiva y analizar los factores asociados al tipo de anticonceptivo usado por las mujeres brasileñas en edad reproductiva. Estudio transversal, de base poblacional, con datos de 19.962 mujeres de 15 a 49 años. Los resultados fueron el uso y el tipo de anticonceptivo, clasificados en: anticonceptivos reversibles de corta duración (SARC), de larga duración (LARC) y permanentes. Las variables explicativas fueron características de la historia reproductiva, sociodemográficas y de acceso a los servicios de salud. Se utilizó la regresión logística multinomial para las estimaciones de la *odds ratio* (OR), siendo los SARC la categoría de referencia. Los análisis fueron realizados en el módulo *survey*, del software Stata, que consideró el efecto del sistema de muestreo complejo de la *Encuesta Nacional de Salud* de 2019. La prevalencia del uso de anticonceptivos fue del 83,7%. Del total de usuarias, 72% usaban SARC, el 23,2% métodos permanentes y el 4,8% LARC. Las mujeres con mayor educación, plan de salud, que tuvieron partos y participaron de grupos de planificación reproductiva tuvieron mayor posibilidad de usar LARC cuando comparados al uso de SARC, mientras que el registro en la unidad básica de salud se asoció con una menor posibilidad de uso. Además, cuanto mayor sea la edad y la paridad, además de vivir con el compañero, mayor será la posibilidad de utilizar métodos permanentes en relación con el uso de SARC. A pesar de la alta cobertura de anticoncepción, la combinación anticonceptiva sigue siendo obsoleta, con un uso predominante de SARC. Además, se observaron importantes desigualdades en el acceso, siendo los LARC accesibles solo para mujeres con mejores condiciones socioeconómicas, mientras que los métodos permanentes se asociaron con un perfil de mayor vulnerabilidad social.

## Introdução

A contracepção constitui aspecto fundamental do desenvolvimento sustentável, uma vez que permite que as pessoas concretizem seus desejos reprodutivos ^1^^,^^2^, o que contribui para a redução da ocorrência de gestações não planejadas, de abortos inseguros e da morbimortalidade materna e infantil ^3^. Também favorece a promoção do crescimento econômico e o empoderamento das mulheres ^1^. Tendo em vista esses, dentre outros benefícios, as Nações Unidas definiram como um dos Objetivos de Desenvolvimento Sustentável (ODS) assegurar, até 2030, o acesso universal à saúde sexual e reprodutiva, incluindo o acesso a contracepção moderna ^2^, evidenciando a relevância do monitoramento contínuo dos indicadores relacionados a essa meta.

O uso de contraceptivos aumentou significativamente em todo o mundo, na medida em que os casais optam cada vez mais por ter menos filhos e que os contraceptivos se tornaram amplamente disponíveis ^3^. Apesar desse aumento, existe muita desigualdade entre e dentro dos países no acesso à contracepção ^3^^,^^‍4^^,^^‍5^. Disparidades em relação a faixa etária, *status* socioeconômico, área de residência, região geográfica, nível de escolaridade ^3^^,^^‍4^^,^^‍5^ e de empoderamento das mulheres ^5^ foram evidenciados em estudos prévios.

No Brasil, houve um importante aumento na prevalência do uso de contraceptivos, seguida de manutenção da cobertura superior a 80% desde 2006 ^6^^,^^‍7^^,^^‍8^. Apesar disso, o acesso à contracepção no país é marcado por fortes desigualdades ^6^^,^^‍7^. Estudos realizados na última década revelam que as mulheres jovens, menos escolarizadas, de classe econômica mais baixa, pretas e pardas e que vivem nas regiões Norte e Nordeste do país são as que apresentam menor prevalência no uso de contraceptivos ^8^^,^^‍9^^,^^‍10^, e também são as mais submetidas à laqueadura ^8^. Já as mulheres brancas, mais escolarizadas e residentes das regiões Sul e Sudeste são as que mais utilizam contraceptivo oral e dupla proteção ^8^. Isso mostra que, para além do acesso, observam-se desigualdades também em relação ao tipo de contraceptivo usado pelas brasileiras ^7^^,^^‍8^.

Destaca-se ainda a manutenção de um *mix* contraceptivo obsoleto no país, desde a década de 1990, caracterizado pela maior prevalência do uso da pílula e da laqueadura em detrimento dos métodos de longa duração, tais como os dispositivos intrauterinos (DIU) e os implantes, usados por menos de 2% das mulheres em 2013 ^7^^,^^‍8^, conforme dados dos inquéritos nacionais, com destaque para a *Pesquisa Nacional de Saúde* (PNS) de 2013. Além disso, mulheres mais jovens e com maior vulnerabilidade social têm menor acesso ao uso de contraceptivos reversíveis de longa duração (LARC) ^11^, o que reforça as iniquidades em relação ao tipo de contraceptivo usado pelas brasileiras e pode sinalizar limitações na escolha dos métodos, principalmente para essas mulheres.

Por outro lado, muitas vezes os estudos sobre contracepção no Brasil - principalmente aqueles em nível nacional ^7^^,^^‍8^ - se limitam à análise da cobertura e a uma abordagem mais descritiva. Além disso, o fato de a cobertura ser alta pode gerar o entendimento equivocado de que o acesso à contracepção no país é universal e não há necessidade de maiores investigações. No entanto, elevadas taxas de gestações não planejadas persistem ^12^, bem como de abortos inseguros ^13^ e de gravidez na adolescência ^14^^,^^‍15^, o que justifica a necessidade do contínuo monitoramento do acesso ao planejamento reprodutivo no país. Ademais, o *mix* se mostrou obsoleto ^7^^,^^‍8^, e os estudos que investigaram fatores associados ao uso de contracepção em nível nacional são aqueles da última *Pesquisa Nacional de Demografia e Saúde* (PNDS), de 2006 ^6^^,^^‍16^, ou locais ^10^^,^^‍17^.

Diante do exposto, o objetivo deste estudo é descrever o *mix* contraceptivo brasileiro com os dados mais recentes da PNS de 2019, além de estimar que fatores estão associados ao tipo de contraceptivo usado pelas brasileiras, considerando-se a classificação dos métodos quanto ao tempo de ação. Uma investigação dos principais grupos de métodos (a depender do tempo de ação, como de curta ou longa duração), bem como dos métodos permanentes, é mais escassa e poderia contribuir para o melhor entendimento do *mix* contraceptivo no país e suas repercussões. Ademais, poderia elucidar diferenças em relação aos fatores que se associam ao uso de LARC e aos métodos permanentes. A continuidade desses estudos também se faz necessária em meio aos cortes orçamentários na área da saúde ^18^ e à perda do espaço da saúde das mulheres nas políticas públicas brasileiras ^8^^,^^‍19^ e, consequentemente, do acesso à contracepção.

## Métodos

Trata-se de estudo epidemiológico transversal que utilizou dados da segunda edição da PNS, de 2019. A PNS é um inquérito de base populacional, representativo da população brasileira, que tem como objetivo fornecer informações sobre os determinantes, condicionantes e necessidades de saúde da população brasileira ^20^, incluindo o planejamento reprodutivo. O inquérito possui desenho amostral complexo, definido por conglomerados em três estágios de seleção: o primeiro, as unidades primárias de amostragem, que podem ser setores censitários ou conjunto de setores; o segundo consistiu na seleção dos domicílios cadastrados no Cadastro Nacional de Endereços para Fins Estatísticos (CNEFE); e o terceiro correspondeu à seleção de um morador do domicílio com 15 ou mais anos de idade ^20^. Em cada estágio, a seleção dos participantes foi por amostragem aleatória simples ^20^. Para maiores detalhes do plano amostral e aspectos metodológicos, outra publicação pode ser consultada ^20^.

**Figura 1:**
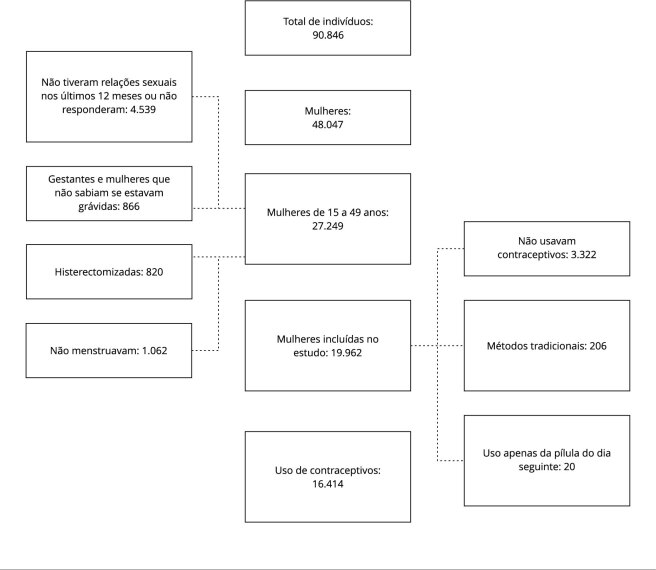
População de estudo.

A população deste estudo foi composta por mulheres de 15 a 49 anos que responderam ao módulo referente à saúde da mulher (Figura 1). Do total de 90.846 indivíduos entrevistados, 48.047 eram mulheres, das quais 27.249 estavam em idade reprodutiva. Dessas mulheres, foram excluídas as gestantes e mulheres que não sabiam se estavam grávidas (n = 866), mulheres histerectomizadas (n = 820), que não menstruavam (n = 1.062) e que não tiveram relações sexuais nos últimos 12 meses ou não quiseram responder a essa questão (n = 4.539), totalizando uma amostra de 19.962 mulheres.

Este estudo tem três desfechos de interesse: o uso de métodos contraceptivos, o tipo de método contraceptivo usado e os grupos de métodos contraceptivos modernos segundo tempo de ação no organismo. A variável uso de contraceptivos foi criada a partir das questões R34 (“A senhora usa algum método para evitar a gravidez atualmente?”), R35 (“Qual o motivo para não evitar a gravidez?”) e R36 (“Que método para evitar a gravidez a sra. usa atualmente?”). O uso de métodos contraceptivos foi categorizado em não (0) e sim (1). Mulheres que responderam que não usavam métodos contraceptivos por terem realizado laqueadura ou vasectomia pelo parceiro foram incluídas na categoria sim.

Para a criação da variável tipo de método contraceptivo usado pelas brasileiras, considerou-se o contraceptivo mais eficaz usado pela mulher ^21^, já que elas poderiam responder que usavam mais de um método. Para isso foi utilizada a variável VDR001 do inquérito; porém, a opção 7 desta variável agrupou vários métodos modernos (camisinha feminina, pílula do dia seguinte, implante, espermicida, diafragma, adesivo e anel), independentemente do seu tempo de ação e eficácia. Diante disso, fizemos a reclassificação dos métodos dessa variável a partir da variável R36 (“Que método para evitar a gravidez a sra. usa atualmente?”) e desagregamos todos os contraceptivos da opção 7. Ressalta-se que todas as análises que usaram o desfecho tipo de método contraceptivo consideraram o método contraceptivo mais eficaz usado pela mulher.

Após a definição de um método para cada mulher, os métodos contraceptivos foram classificados quanto ao tempo de ação no organismo em: (1) contraceptivos reversíveis de curta duração (SARC) (pílula, preservativo masculino e feminino, diafragma, injetável, creme/óvulo adesivo e anel); (2) LARC (DIU e implantes); e (3) permanentes (laqueadura e vasectomia). Para a análise do desfecho tipo de método contraceptivo, as mulheres que usavam apenas “tabelinha” (n = 167), outros métodos contraceptivos tradicionais (n = 39) ou relataram apenas o uso da pílula do dia seguinte como método contraceptivo (n = 20) e as mulheres que não usavam método contraceptivo (n = 3.322) foram excluídas, totalizando 16.414 usuárias de contracepção moderna (Figura 1).

As variáveis explicativas incluíram: características da história reprodutiva (número de partos: nenhum, um a dois e três ou mais); acesso aos serviços de saúde (participação em grupo de planejamento reprodutivo, ser cadastrada na unidade básica de saúde - UBS - e ter plano de saúde - categorizadas em sim e não); e características sociodemográficas (local de moradia: urbano e rural; região: Norte, Nordeste, Centro-oeste, Sudeste e Sul; faixa etária: 15 a 24 anos, 25 a 34 anos e 35 a 49 anos; escolaridade: 0 a 9 anos de estudo, 10 a 12 anos e 13 anos ou mais; raça/cor: branca, preta e parda; situação conjugal: vive com companheiro e não vive com companheiro; renda: rendimento domiciliar *per capita*, em salários mínimos - até 1, de 1 a 3 e mais que 3; trabalho remunerado - sim e não). Para definição do valor do salário mínimo, que corresponde à remuneração mínima do trabalhador fixada por lei, considerou-se o valor em vigor no mês de referência da pesquisa ^22^.

Primeiramente, a prevalência do uso e do tipo de contraceptivo usado pelas brasileiras foram estimadas com seus respectivos intervalos de 95% de confiança (IC95%). Em seguida, a prevalência do tipo de método classificado quanto ao tempo de ação, incluindo a categoria de não usuárias, foi estimada segundo as características da história reprodutiva, do acesso aos serviços de saúde e sociodemográficas.

Depois, foi utilizado o modelo de regressão logística multinomial para estimar a *odds ratio* (OR) não ajustada com os IC95% de cada variável explicativa com o tipo de método classificado quanto ao tempo de ação, desfecho principal do estudo. A categoria de referência foi: mulheres que usavam SARC. A seguir, as variáveis com valor de p < 0,20 foram inseridas no modelo multivariado. Utilizou-se o critério *forward*, sendo as variáveis de nível mais proximal (história reprodutiva) inseridas primeiro, seguidas pelas variáveis do nível intermediário (acesso aos serviços de saúde) e, por último, as variáveis do nível distal (características sociodemográficas). As variáveis com valor de p > 0,05 foram retiradas do modelo, utilizando-se o critério *backward*. Destaca-se que o modelo multinomial gerou a estimativa de dois valores de OR: o primeiro comparando os métodos de longa duração com os de curta duração, e o segundo comparando os métodos permanentes com os de curta duração. Utilizou-se o teste Wald em cada entrada de variável no modelo, e também após o modelo final, para avaliar a contribuição de cada variável na modelagem ^23^.

Também foi avaliada a relação entre as variáveis explicativas para inserção no modelo multivariado. Utilizou-se análise de distribuição entre os pares de variáveis explicativas e o teste qui-quadrado de Pearson nessa etapa. Observou-se que as mulheres com maior renda eram as que tinham maior escolaridade (82,8%) e acesso a plano de saúde (81,4%), com significância estatística das diferenças entre as proporções (p < 0,0001). Assim, no modelo final, optou-se pela manutenção das variáveis escolaridade e plano de saúde, considerando-se a escolaridade como *proxy* de renda e com alta taxa de resposta, e o plano de saúde como um determinante importante do acesso aos LARC, conforme reportado por alguns estudos ^24^^,^^‍25^.

Os dados foram analisados com auxílio do software Stata (https://www.stata.com), versão 15.0, considerando-se o nível de 5% de significância. As estimativas foram realizadas no módulo *survey*, que considerou na análise o efeito do plano amostral da PNS (estrato, conglomerados e pesos individuais), com o objetivo de produzir estimativas populacionais para a subpopulação de mulheres em idade reprodutiva ^23^^,^^‍26^.

A PNS foi aprovada pela Comissão Nacional de Ética em Pesquisa do Conselho Nacional de Saúde sob o parecer nº 3.529.376, em 23 de agosto de 2019. A participação na pesquisa foi voluntária, a confidencialidade das informações garantida e todos os participantes assinaram o Termo de Consentimento para a Entrevista ^20^. Os dados da PNS estão publicamente disponíveis na página da Internet do Instituto Brasileiro de Geografia e Estatística ^27^.

## Resultados

A maior proporção das mulheres de 15 a 49 anos vivia em áreas urbanas (87,6%), nas regiões Sudeste (42,5%) e Nordeste (26,2%), tinha mais de 35 anos (45,9%), de 10 a 12 anos de estudo (46,5%), era parda (46,1%), com renda de até um salário mínimo (58%) e trabalho remunerado (73,3%), vivia com companheiro (66,4%), já teve partos (68,6%), não participou de grupos de planejamento reprodutivo (95,4%), não tinha plano de saúde (72,7%) e era cadastrada na UBS (60,7%) (Tabela 1).

**Tabela 1:** Características das mulheres brasileiras de 15 a 49 anos. *Pesquisa Nacional de Saúde* de 2019 (PNS 2019).

Características	%	IC95%
Local de residência		
Rural	12,4	11,7; 13,2
Urbano	87,6	86,8; 88,3
Região de moradia		
Norte	8,6	8,1; 9,1
Nordeste	26,2	25,1; 27,3
Centro-oeste	8,1	7,5; 8,7
Sudeste	42,5	40,8; 44,2
Sul	14,7	13,9; 15,6
Faixa etária (anos)		
15 a 24	22,9	21,7; 24,2
25 a 34	31,1	30,0; 32,3
35 a 49	45,9	44,6; 47,2
Escolaridade (anos de estudo)		
0 a 9	26,0	24,9; 27,2
10 a 12	46,5	45,2; 47,7
13 ou mais	27,5	26,3; 28,7
Raça/Cor		
Branca	41,8	40,5; 43,2
Preta	11,4	10,6; 12,2
Parda	46,7	45,5; 48,0
Trabalho remunerado		
Não	26,6	25,3; 28,0
Sim	73,3	72,0; 74,6
Renda (salários mínimos)		
Até 1	58,0	56,5; 59,4
1 a 3	32,9	31,6; 34,2
Mais que 3	9,1	8,3; 9,9
Situação conjugal		
Não vive com companheiro	33,6	32,4; 34,9
Vive com companheiro	66,4	65,1; 67,6
Número de partos		
Nunca teve	31,4	30,1; 32,8
1 a 2	49,1	47,8; 50,4
3 ou mais	19,5	18,6; 20,4
Grupo de planejamento reprodutivo		
Não	95,4	94,9; 95,9
Sim	4,5	4,1; 5,1
Plano de saúde		
Não	72,7	71,4; 73,9
Sim	27,3	26,1; 28,6
Cadastrada na UBS		
Não	39,3	37,8; 40,8
Sim	60,7	59,2; 62,2

IC95%: intervalo de 95% de confiança; UBS: unidade básica de saúde.

A prevalência do uso de contraceptivos foi de 83,7% (IC95%: 82,7; 84,7) (dado não mostrado). Do total de usuárias, os métodos mais utilizados pelas brasileiras eram: pílula (40,6%), preservativo masculino (20,3%), laqueadura (17,3%) e injetáveis (9,8%) (Tabela 2). Ao classificar os métodos pelo tempo de ação no organismo, observamos uma alta prevalência de uso de SARC (72%), quando comparado aos LARC (4,8%) e métodos permanentes (23,2%) (Tabela 2).

**Tabela 2:** Prevalência do tipo de contraceptivo usados pelas mulheres brasileiras em idade reprodutiva. *Pesquisa Nacional de Saúde* de 2019 (PNS 2019).

Métodos contraceptivos	Prevalência	IC95%
Métodos de curta duração (SARC)	72,0	70,8; 73,1
Pílula	40,6	39,4; 41,8
Preservativo masculino	20,3	19,2; 21,5
Injetáveis	9,8	8,9; 10,8
Outros métodos contraceptivos modernos	0,4	0,3; 0,6
Métodos de longa duração (LARC)	4,8	4,3; 5,4
DIU	4,4	3,9; 4,9
Implante	0,4	0,3; 0,6
Métodos permanentes	23,2	22,2; 24,2
Laqueadura	17,3	16,4; 18,2
Vasectomia	5,5	5,0; 6,2
Métodos tradicionais	1,2	0,9; 1,58

IC95%: intervalo de 95% de confiança; LARC: contraceptivos reversíveis de longa duração; SARC: contraceptivos reversíveis de curta duração.

Nota: o total de SARC, LARC e métodos permanentes não corresponde ao total de cada tipo de método, pois para essa estimativa foram contabilizadas todas as usuárias de métodos contraceptivos. A classificação dos métodos contraceptivos quanto ao tempo de ação não incluiu usuárias de métodos contraceptivos tradicionais.

Quanto ao tipo de método usado, observou-se maior proporção de uso de SARC pelas mulheres que viviam nas regiões Sul (66,7%) e Sudeste (61,7%), que tinham entre 15 e 24 anos (77,7%) e que não viviam com companheiro (73%) (Tabela 3). Em relação ao uso de LARC, observou-se maior proporção de uso para mulheres que viviam em áreas urbanas (4,4%) e na Região Sul (4,9%), tinham maior escolaridade (7,2%), trabalho remunerado (4,7%), maior renda (9,8%), plano de saúde (8,3%) e não eram cadastradas na UBS (5,2%) (Tabela 3). Quanto ao uso de métodos permanentes, observou-se maior prevalência entre as mulheres que viviam em áreas rurais (25,4%), nas regiões Norte (24,1%), Nordeste (23,5%) e Centro-oeste (24,5%), de maior faixa etária (32,9%), menor escolaridade (30,1%), que não trabalhavam (22,8%), que tinham renda de até 1 salário mínimo (22,3%), que viviam com companheiro (24,9%), que não tinham plano de saúde (20,5%), não participaram de grupos de planejamento reprodutivo (19,6%) e tinham cadastro na UBS (21,1%) (Tabela 3).

**Tabela 3:** Prevalência do tipo de contraceptivo usado pelas mulheres de 15 a 49 anos, segundo características sociodemográficas, reprodutivas e de acesso aos serviços de saúde, Brasil. *Pesquisa Nacional de Saúde* de 2019 (PNS 2019).

Características	Não usa método contraceptivo	SARC	LARC	Permanentes
	% (IC95%)	% (IC95%)	% (IC95%)	% (IC95%)
Local de residência				
Rural	15,7 (14,0; 17,6)	57,1 (54,5; 59,6)	1,8 (1,2; 2,8)	25,4 (23,2; 27,7)
Urbano	16,6 (15,5; 17,7)	60,5 (59,2; 61,8)	4,4 (3,9; 4,9)	18,5 (17,6; 19,5)
Região de moradia				
Norte	19,4 (17,4; 21,6)	54,1 (51,4; 56,8)	2,4 (1,5; 3,8)	24,1 (22,0; 26,3)
Nordeste	16,4 (15,1; 17,9)	57,1 (55,3; 58,9)	3,0 (2,4; 3,6)	23,5 (22,1; 25,0)
Centro-oeste	15,1 (13,3; 17,0)	56,0 (53,1; 58,7)	4,5 (3,5; 5,7)	24,5 (22,1; 27,1)
Sudeste	16,5 (14,6; 18,6)	61,7 (59,3; 64,0)	4,7 (3,8; 5,7)	17,1 (15,5; 18,8)
Sul	15,5 (13,6; 17,5)	66,7 (64,4; 69,0)	4,9 (4,0; 6,0)	12,9 (11,2; 14,8)
Faixa etária (anos)				
15 a 24	18,7 (16,3; 21,4)	77,7 (74,9; 80,3)	2,5 (1,8; 3,4)	1,0 (0,7; 1,5)
25 a 34	15,0 (13,7; 16,3)	67,0 (65,2; 68,8)	4,9 (4,1; 5,9)	13,0 (11,7; 14,5)
35 a 49	16,3 (15,0; 17,8)	46,5 (44,9; 48,2)	4,2 (3,6; 4,9)	32,9 (31,3; 34,6)
Escolaridade (anos de estudo)				
0 a 9	18,2 (16,3; 20,4)	50,2 (48,0; 52,5)	1,4 (1,1; 1,9)	30,1 (28,1; 32,2)
10 a 12	15,6 (14,2; 17,1)	63,6 (61,9; 65,3)	3,6 (3,0; 4,4)	17,1 (15,8; 18,4)
13 ou mais	16,2 (14,7; 17,9)	63,4 (61,3; 65,5)	7,2 (6,2; 8,4)	13,1 (11,8; 14,6)
Raça/Cor				
Branca	16,7 (15,1; 18,6)	61,8 (59,7; 63,9)	5,3 (4,4; 6,2)	16,2 (14,8; 17,6)
Preta	16,0 (13,4; 19,0)	60,8 (57,1; 64,4)	4,2 (3,0; 5,8)	19,0 (16,6; 21,7)
Parda	16,4 (15,1; 17,7)	58,2 (56,4; 59,9)	3,0 (2,6; 3,5)	22,5 (21,1; 23,9)
Trabalho remunerado				
Não	16,9 (15,5; 18,5)	57,9 (55,8; 59,9)	2,4 (1,9; 3,0)	22,8 (21,2; 24,5)
Sim	16,3 (15,1; 17,6)	60,9 (59,5; 62,3)	4,7 (4,1; 5,3)	18,1 (17,1; 19,2)
Renda (salários mínimos)				
Até 1	16,6 (15,2; 18,2)	58,4 (56,8; 60,0)	2,6 (2,2; 3,1)	22,3 (21,1; 23,6)
1 a 3	15,2 (13,7; 16,7)	63,3 (61,2; 65,3)	5,0 (4,2; 6,0)	16,5 (15,0; 18,1)
Mais que 3	20,3 (17,4; 23,4)	59,2 (55,7; 62,7)	9,8 (7,9; 12,0)	10,7 (8,8; 13,0)
Situação conjugal				
Não vive com companheiro	15,1 (13,5; 16,8)	73,0 (71,0; 75,0)	3,4 (2,8; 4,2)	8,4 (7,6; 9,4)
Vive com companheiro	17,2 (16,1; 18,3)	53,5 (52,1; 54,9)	4,4 (3,8; 5,0)	24,9 (23,8; 26,2)
Número de partos				
Nunca teve	22,7 (20,8; 24,8)	71,0 (68,8; 73,0)	2,6 (2,0; 3,4)	3,7 (3,0; 4,5)
1 a 2	14,8 (13,7; 16,0)	61,5 (59,9; 63,1)	5,7 (5,0; 6,5)	17,9 (16,8; 19,2)
3 ou mais	10,5 (8,7; 12,6)	39,1 (36,5; 41,7)	2,1 (1,5; 3,0)	48,3 (45,6; 50,9)
Grupo de planejamento reprodutivo				
Não	16,5 (15,5; 17,6)	59,9 (58,7; 61,2)	3,9 (3,5; 4,4)	19,6 (18,7; 20,5)
Sim	15,2 (11,5; 20,0)	63,6 (58,5; 68,4)	6,2 (4,0; 9,4)	14,9 (11,9; 18,6)
Plano de saúde				
Não	16,7 (15,6; 18,0)	60,2 (58,9; 61,6)	2,5 (2,1; 3,0)	20,5 (19,5; 21,6)
Sim	15,7 (14,2; 17,4)	59,8 (57,5; 62,1)	8,3 (7,2; 9,6)	16,2 (14,6; 17,9)
Cadastrada na UBS				
Não	18,9 (17,4; 20,5)	59,1 (57,2; 61,1)	5,2 (4,5; 6,2)	16,7 (15,3; 18,2)
Sim	14,9 (13,7; 16,2)	60,7 (59,2; 62,2)	3,3 (2,8; 3,8)	21,1 (19,9; 22,3)

IC95%: intervalo de 95% de confiança; LARC: contraceptivos reversíveis de longa duração; SARC: contraceptivos reversíveis de curta duração; UBS: unidade básica de saúde.

Quanto aos fatores associados ao tipo de método usado pelas brasileiras, observamos nas análises não ajustadas que a chance de usar LARC em relação aos SARC foi maior para mulheres que viviam em áreas urbanas, nas regiões Sudeste e Centro-oeste, que tinham trabalho remunerado, viviam com companheiro, tinham plano de saúde e histórico de um a dois partos anteriores. Destaca-se que, quanto maior a faixa etária, a escolaridade e a renda, maior a chance de usar LARC quando comparados aos SARC (Tabela 4). Por outro lado, observou-se menor chance de uso desses métodos para mulheres que tinham cadastro na UBS. No modelo multivariado, as variáveis que permaneceram associadas foram escolaridade, número de partos, ter plano de saúde, cadastro na UBS e ter participado de grupos de planejamento reprodutivo (p < 0,05) (Tabela 4).

**Tabela 4:** Fatores associados ao tipo de contraceptivo adotado pelas mulheres brasileiras de 15 a 49 anos, Brasil. *Pesquisa Nacional de Saúde* de 2019 (PNS 2019).

Características	**LARC *vs*. SARC**	**Permanentes *vs*. SARCS**	**LARC *vs*. SARC**	**Permanentes *vs*. SARC**
	OR (IC95%)	OR (IC95%)	OR ajustado (IC95%)	OR ajustado (IC95%)
Local de residência				
Rural	Referência	Referência		
Urbano	2,25 (1,45; 3,47)	0,69 (0,60; 0,79)		
Região de moradia				
Norte	Referência	Referência	Referência	Referência
Nordeste	1,18 (0,70; 1,97)	0,92 (0,79; 1,07)	1,20 (0,72; 2,00)	0,88 (0,74; 1,05)
Centro-oeste	1,82 (1,06; 3,11)	0,98 (0,81; 1,19)	1,42 (0,83; 2,43)	1,06 (0,85; 1,31)
Sudeste	1,72 (1,03; 2,88)	0,62 (0,52; 0,74)	1,20 (0,71; 2,03)	0,61 (0,50; 0,75)
Sul	1,67 (1,00; 2,80)	0,43 (0,35; 0,53)	1,28 (0,76; 2,17)	0,38 (0,30; 0,48)
Faixa etária (anos)				
15 a 24	Referência	Referência	Referência	Referência
25 a 34	2,29 (1,59; 3,30)	14,47 (9,93; 21,07)	1,38 (0,91; 2,08)	7,80 (5,35; 11,38)
35 a 49	2,81 (1,97; 4,01)	52,61 (36,84; 75,12)	1,44 (0,95; 2,19)	23,78 (16,41; 34,47)
Escolaridade (anos de estudo)				
0 a 9	Referência	Referência	Referência	Referência
10 a 12	2,02 (1,44; 2,82)	0,45 (0,39; 0,51)	1,94 (1,35; 2,81)	0,87 (0,74; 1,03)
13 ou mais	4,01 (2,89; 5,56)	0,34 (0,30; 0,41)	2,85 (1,90; 4,28)	0,71 (0,58; 0,88)
Raça/Cor				
Preta	Referência	Referência		
Branca	1,24 (0,84; 1,83)	0,84 (0,68; 1,03)		
Parda	0,75 (0,50; 1,12)	1,24 (1,02; 1,50)		
Trabalho remunerado				
Não	Referência	Referência		
Sim	1,87 (1,41; 2,46)	0,75 (0,67; 0,85)		
Renda (salários mínimos)				
Até 1	Referência	Referência		
1 a 3	1,77 (1,35; 2,31)	0,68 (0,59; 0,78)		
Mais que 3	3,67 (2,71; 4,97)	0,47 (0,37; 0,60)		
Situação conjugal				
Não vive com companheiro	Referência	Referência	Referência	Referência
Vive com companheiro	1,72 (1,34; 2,22)	4,04 (3,52; 4,65)	1,13 (0,86; 1,49)	2,51 (2,15; 2,93)
Número de partos				
Nunca teve	Referência	Referência	Referência	Referência
1 a 2	2,51 (1,86; 3,39)	5,64 (4,51; 7,06)	2,64 (1,85; 3,77)	2,16 (1,71; 2,74)
3 ou mais	1,49 (0,97; 2,29)	23,89 (18,79; 30,36)	2,27 (1,41; 3,66)	7,09 (5,42; 9,29)
Grupo de planejamento reprodutivo				
Não	Referência	Referência	Referência	Referência
Sim	1,48 (0,93; 2,36)	0,72 (0,55; 0,95)	1,74 (1,08; 2,79)	0,65 (0,46; 0,91)
Plano de saúde				
Não	Referência	Referência	Referência	Referência
Sim	3,38 (2,64; 4,33)	0,79 (0,68; 0,92)	2,45 (1,79; 3,36)	1,03 (0,85; 1,25)
Cadastrada na UBS				
Não	Referência	Referência	Referência	Referência
Sim	0,61 (0,48; 0,77)	1,23 (1,07; 1,40)	0,71 (0,55; 0,90)	1,00 (0,86; 1,16)

IC95%: intervalo de 95% de confiança; LARC: contraceptivos reversíveis de longa duração; OR: *odds ratio*; SARC: contraceptivos reversíveis de curta duração; UBS: unidade básica de saúde.

Nas análises não ajustadas para o uso de métodos permanentes observou-se que quanto maior a faixa etária e maior o número de partos, maior a chance de uso em relação aos SARC. Além disso, mulheres pardas, que tinham cadastro na UBS e viviam com companheiro também tinham maior chance de uso de métodos permanentes se comparadas às usuárias de SARC. Observou-se ainda que possuir maior escolaridade e renda, viver em áreas urbanas e nas regiões Sul e Sudeste, ter trabalho remunerado e plano de saúde e haver participado de grupos de planejamento reprodutivo foram associados a menor chance de uso de métodos permanentes quando comparados aos SARC. No modelo multivariado, as variáveis que permaneceram associadas foram faixa etária, paridade, região de moradia, escolaridade, participação em grupo de planejamento reprodutivo e viver com companheiro (p < 0,05) (Tabela 4).

## Discussão

Os resultados do presente estudo evidenciam a manutenção de uma elevada prevalência de uso de contraceptivos pelas mulheres brasileiras, superior a 80%, conforme observado em estudos prévios ^7^^,^^‍8^. Porém, mantém-se um *mix* contraceptivo obsoleto, com mais de 60% das mulheres usando pílula ou preservativo, 17% das mulheres esterilizadas e apenas 5% usando LARC, corroborando achados de outros estudos (que incluíram somente mulheres de 18 a 49 anos), com poucas mudanças em relação a 2013 ^7^^,^^‍8^.

Nesse sentido, destaca-se que altas prevalências de uso de contraceptivos não se traduzem necessariamente em um *mix* contraceptivo diversificado, como também foi observado em países como a China e Vietnã, que têm altas prevalências de contracepção, apesar do uso concentrado em um ou dois métodos ^28^. Esses resultados podem refletir limitações na escolha do método mais adequado de acordo com a necessidade de cada mulher, como observado no Brasil. Além disso, observamos que mais de 70% das usuárias de contracepção usam SARC, sendo a pílula e o preservativo os métodos mais usados e que possuem as maiores taxas de falha - 9% e 18%, respectivamente ^29^ -, o que pode contribuir para as elevadas taxas de gestação não planejada ^12^ e abortos ^13^, conforme observado em países como Estados Unidos ^30^ e França ^31^.

Nosso estudo também mostrou que mulheres mais jovens, nulíparas e sem cadastro nas UBS apresentaram menor prevalência de uso de contraceptivos, o que pode contribuir para a elevada ocorrência de gestações na adolescência no país ^14,‍15^. Sabe-se que, no Brasil e em vários países de baixa e média renda, a maioria das gestações ocorre antes de qualquer uso de contraceptivo, o que repercute em uma proporção grande de mulheres com menos de 30 anos atingindo o número de filhos desejado e com um período longo para evitar um filho extra ^7^. Isso ocorre por falta de acesso a contracepção eficaz e informações em tempo oportuno, principalmente na adolescência ^7^, fato corroborado pelos achados deste estudo.

Nossos resultados também apontam para um possível problema de disponibilidade de métodos contraceptivos, visto que os métodos mais usados, como a pílula e o preservativo, também são os mais disponíveis nas UBS, enquanto o DIU é o método menos disponível ^24^ e menos utilizado pelas brasileiras, o que também foi demonstrado em estudo prévio ^24^: ou seja, o uso de método contraceptivo depende mais da oferta do que da procura. Assim, caso o método contraceptivo não esteja disponível ou não seja oferecido, isso determinará a sua não utilização. Além disso, mulheres sem cadastro na UBS também apresentam menor prevalência de uso de métodos contraceptivos, ressaltando a importância desse provedor para o acesso a contracepção no país.

Por outro lado, uma avaliação com provedores de contracepção moderna em países da América Latina, Ásia e África mostrou que entre 40% e 49% das usuárias de métodos contraceptivos modernos obtiveram seus métodos por meio do setor privado, sendo que o aumento do uso de contraceptivos observado entre 1992 e 2012 foi impulsionado pelo aumento do uso de SARC ^32^. Ainda, estudo que avaliou aspectos relacionados ao acesso a contraceptivos orais e injetáveis no Brasil mostrou que a maioria das mulheres obtinham esses medicamentos com recursos próprios nas farmácias ^9^. Esses resultados corroboram nossos achados ao mostrar que os SARC são mais acessíveis que os LARC, que, apesar de ter seu uso duplicado em relação a 2013 ^8^, ainda apresentam número muito incipiente de usuárias.

O aumento do uso de LARC, mais especificamente do DIU, provavelmente está relacionado à regulamentação da inserção do DIU após o parto e abortamento nas maternidades ^33^. Nossos resultados mostraram que mulheres que tiveram um ou mais partos tinham maior chance de usar LARC em relação a SARC quando comparadas com as mulheres que nunca tiveram partos, corroborando essa hipótese. Em alguns estados brasileiros - como São Paulo, Rio Grande do Sul e Ceará - foi instituída legislação para promover o uso de LARC entre as mulheres mais vulneráveis, incluindo usuárias de drogas, mulheres em situação de rua e adolescentes, o que pode favorecer a provisão seletiva de LARC em um contexto de coerção contraceptiva ^34^.

O presente estudo também evidenciou iniquidades em relação ao acesso aos LARC, uma vez que as mulheres com maior vulnerabilidade social (residentes em áreas rurais, com menor renda e escolaridade, sem acesso a plano de saúde e trabalho remunerado) apresentaram menor prevalência de uso desses métodos. Esses achados foram confirmados no modelo final, com maior chance de uso desses métodos contraceptivos por aquelas com melhores condições socioeconômicas, tais como maior escolaridade e acesso ao plano de saúde, corroborando achados de outros estudos ^35^^,^^36^. Sabe-se que a relação entre maior escolaridade e fecundidade tem um impacto duradouro na vida das mulheres, pois constitui-se em recurso de maior acesso à informação e conhecimento, além de ser veículo de mobilidade socioeconômica (e, consequentemente, autonomia) e modificador de atitudes que influenciam o comportamento contraceptivo, incluindo a escolha do método mais adequado à sua necessidade ^37^^,^^38^. Resultados semelhantes foram observados na América Latina e Caribe, onde os LARC são usados por menos de 10% das mulheres ^11^.

Acrescenta-se a esse cenário a menor disponibilidade do DIU no setor público nesses países, onde as mulheres com maior vulnerabilidade social usualmente acessam os contraceptivos ^24^ - o que também foi observado em nosso estudo, visto que mulheres sem cadastro na UBS apresentaram menor prevalência e menor chance de usar LARC. Também foi observada menor prevalência desses métodos entre mulheres mais jovens e nulíparas, corroborando estudos prévios que mostraram que essas mulheres enfrentam maiores barreiras de acesso aos LARC ^39^^,^^40^. Assim, reforça-se a hipótese de acesso limitado a uma ampla gama de contraceptivos, principalmente nos serviços públicos de saúde e para essas mulheres.

Outro fator associado a maior chance de usar LARC foi a participação em grupos de planejamento reprodutivo, o que reflete a importância de conhecer as opções de métodos contraceptivos disponíveis para uma decisão livre e informada a respeito da melhor opção contraceptiva para cada mulher. A baixa prevalência de acesso a esses grupos reforça a baixa disponibilidade de aconselhamento - importante prática para garantia desses direitos, para além do acesso ao método contraceptivo.

Por conseguinte, destaca-se que a educação em saúde sexual e reprodutiva constitui-se como um dos pilares para a promoção do uso de contraceptivos, especialmente dos LARC e para o grupo de mulheres mais jovens, incluindo adolescentes ^41^. Também pode promover a autonomia para a escolha livre e informada do contraceptivo mais adequado à necessidade de cada mulher ^42^. Nos Estados Unidos, por exemplo, algumas intervenções em larga escala foram eficazes na promoção do uso de LARC entre adolescentes e mulheres jovens, tal como reportado pelo Projeto CHOICE ^42^. Também foram feitas intervenções em parcerias com escolas, como os centros de saúde de base escolar, onde são ofertados métodos contraceptivos, incluindo LARC ^43^.

Por fim, houve uma redução da prevalência de realização da laqueadura e um discreto aumento da vasectomia em relação a 2013, considerando-se mulheres de 18 a 49 anos. Ademais, observamos um perfil de maior vulnerabilidade social para mulheres que usaram esses métodos contraceptivos, o que pode estar relacionado à fecundidade alta em idades mais jovens, bem como a dificuldades de acessar outros contraceptivos mais eficazes ^6^^,^^‍7^ e com menos efeitos colaterais ^44^. Observamos ainda que, quanto maior a idade e paridade, associadas ao fato de viver com o companheiro, maior foi a chance desse tipo de contracepção quando comparado ao uso de SARC, o que pode ser explicado pelo atingimento da fecundidade desejada e pela necessidade de um método eficaz para evitar nova gestação ^44^. Estudo que avaliou o papel dos métodos permanentes em países de baixa e média renda mostrou que eles são responsáveis por uma alta proporção de demanda por planejamento familiar satisfeita, sendo observada maior prevalência em alguns subgrupos de mulheres mais vulneráveis ^45^, o que corrobora nossos achados.

Uma preocupação em relação às altas prevalências de métodos permanentes em mulheres com maior vulnerabilidade social refere-se ao quão bem informadas essas mulheres estão sobre a natureza permanente do método e sobre a disponibilidade de outros contraceptivos modernos, como os LARC, que têm eficácia semelhante a esses métodos ^29^, evidenciando-se a necessidade de aconselhamento contraceptivo qualificado ^45^. Nesse sentido, nossos resultados também mostraram que as mulheres mais escolarizadas, residentes nas regiões Sul e Sudeste, bem como as mulheres que participaram de grupos de planejamento reprodutivo, tiveram menor chance de usar métodos permanentes quando comparados aos SARC, o que pode indicar que essas mulheres têm mais acesso a e conhecimento sobre outras opções de contracepção.

Finalmente, destaca-se, como um dos nossos principais resultados sobre a associação de fatores sociodemográficos ao uso de LARC e métodos permanentes, que mulheres com melhores condições socioeconômicas têm maior chance de usar LARC e menor chance de usar métodos permanentes, o que evidencia a manutenção de importante desigualdade social no acesso a contracepção no país, conforme observado em estudos prévios, de caráter descritivo ^7^^,^^‍8^^,^^‍11^. Um dos principais fatores que pode ter contribuído para essa desigualdade foi a rapidez com que a transição da fecundidade ocorreu no país na ausência de programas de planejamento reprodutivo bem estruturados, em um contexto de ilegalidade na provisão da laqueadura e de uso indiscriminado da pílula ^7^. 

Apesar dos avanços das políticas nesse âmbito a partir da década de 1980, nos últimos anos temos observado cortes no orçamento da saúde ^18^, bem como a extinção de algumas políticas públicas de saúde da mulher ^46^ - além da pandemia de COVID-19, que tanto piorou a desigualdade social como limitou o acesso aos serviços de planejamento reprodutivo no país ^25^, o que pode comprometer os avanços alcançados até o momento. Soma-se a isso a onda de conservadorismo no país ^47^, que tem contribuído para retrocessos como a não assinatura do documento da Organização Mundial da Saúde (OMS) que firma compromissos acerca da saúde sexual e reprodutiva das populações junto aos demais países ^19^, e a campanha sobre prevenção da gravidez na adolescência, que prioriza a abstinência sexual em detrimento da promoção do uso de contraceptivos ^48^.

### Limitações

Algumas questões relacionadas ao questionário da PNS precisam ser corrigidas para permitir melhor monitoramento desses indicadores no país. O uso atual de contraceptivos não está bem definido na pergunta correspondente, que vem depois de outra sobre atividade sexual nos últimos 12 meses; pode-se inferir que este seja o período de referência. A laqueadura e a vasectomia não foram incluídas como métodos contraceptivos, e sim como motivos para não se evitar uma gestação, o que pode levar a uma subestimação do uso desses métodos. Além disso, o inquérito excluiu questões sobre aborto e idade da primeira gestação e não incluiu questões sobre o desejo de engravidar, um dos determinantes do uso de contraceptivos. Por outro lado, o inquérito de 2019 avança em relação ao de 2013 ao incluir mulheres a partir de 15 anos e ao considerar o método mais eficaz usado pela mulher - apesar de agrupar o implante em uma categoria de métodos de curta duração, o que foi corrigido neste estudo.

## Conclusão

Nossos achados mostraram que o *mix* contraceptivo no país se mantém obsoleto, com alta prevalência de SARC, principalmente entre mulheres mais jovens e nulíparas. Também evidenciou os fatores associados ao tipo de contraceptivo usado pelas mulheres brasileiras, demonstrando que estes se associam de forma diferente em relação ao uso de LARC e métodos permanentes.

Ressalta-se a importância do monitoramento dos indicadores de contracepção no país, visto que constituem ferramenta de avaliação e implementação de políticas públicas de planejamento reprodutivo. Mesmo com uma alta cobertura de uso de contracepção, a desigualdade persiste evidenciando a necessidade de ampliação do acesso a métodos mais eficazes, principalmente nos serviços públicos de saúde e para mulheres mais jovens e com maior vulnerabilidade social, de maneira a tornar possível a meta da Agenda 2030 de “não deixar ninguém para trás”.

Por fim, nossos achados apontam para a necessidade de um aconselhamento contraceptivo qualificado para que as mulheres possam fazer uma escolha livre e informada do contraceptivo mais adequado a sua necessidade, respeitando-se, assim, os direitos sexuais e reprodutivos.
